# Case Report of Clade Ib Monkeypox Virus Infection Linked to Travel to Democratic Republic of the Congo, Thailand, 2024

**DOI:** 10.3201/eid3108.250255

**Published:** 2025-08

**Authors:** Drunphob Srithammavong, Chanunya Srihawan, Rossaphorn Kittiyaowamarn, Rapeepong Suphanchaimat, Thitipong Yingyong, Wichan Bunyakitikorn

**Affiliations:** Ministry of Public Health, Bangkok, Thailand (D. Srithammavong, R. Kittiyaowamarn, R. Suphanchaimat, T. Yingyong, W. Bunyakitikorn); Samitivej Sukhumvit Hospital, Bangkok (C. Srihawan)

**Keywords:** mpox, monkeypox virus, clade Ib, real-time PCR, public health, sexually transmitted infections, viruses, zoonoses, Democratic Republic of the Congo, Thailand

## Abstract

We report clade Ib monkeypox virus infection in a patient who returned to Thailand from the Democratic Republic of the Congo, the subclade epicenter. Improved diagnostic testing, public health response, and surveillance systems for mpox are needed in Thailand, and preexposure mpox vaccination should be considered, especially for high-risk persons.

Mpox is an infectious disease caused by monkeypox virus (MPXV), which is primarily transmitted through close contact with infected persons (*1*). In late 2023, a novel clade Ib MPXV was identified in the Democratic Republic of the Congo (DRC) after earlier mpox outbreaks in the United States in 2003 and the global outbreak in 2022 (*2*,*3*). Because of the rise in clade I–associated mpox cases, the World Health Organization (WHO) declared the outbreak in Africa a Public Health Emergency of International Concern in May 2024 (*4*). We report a case of clade Ib MPXV–associated mpox in Thailand and highlight the challenges in mpox public health responses. The case investigation was conducted by authorized public health officers according to the Communicable Disease Act of Thailand. Patient information remains confidential.

## The Study

A 66-year-old man of German nationality who resides in eastern Thailand traveled to Germany on June 18, 2024. On July 30, he departed for Rwanda and then traveled to Bukavu, South Kivu, DRC, on August 1. He stayed with 2 friends in an apartment and denied participating in any sexual activity or having contact with infected persons during his visit. His friends reported no abnormal symptoms. He reported that he rarely wore a mask or washed his hands with soap or sanitizer during his stay. On August 10, genital itching developed. On August 13, his co-worker drove him to the border, and he took a taxi to the airport in Rwanda, where he departed and transited through Qatar, arriving in Thailand on August 14. On that same day, his symptoms progressed to fever, muscle aches, sore throat, fatigue, and rash, which primarily affected his genitalia, trunk, extremities, and face. His wife picked him up from Suvarnabhumi Airport (Thailand); they had dinner together in Bangkok before checking into a hotel. On August 15, he visited a private hospital and was admitted. On examination, he had multiple discrete erythematous maculopapular lesions and a few vesicular lesions distributed across his face, trunk, and extremities and had some necrotic papules with overlying scabs on his penis and right thigh (Figure).

**Figure:**
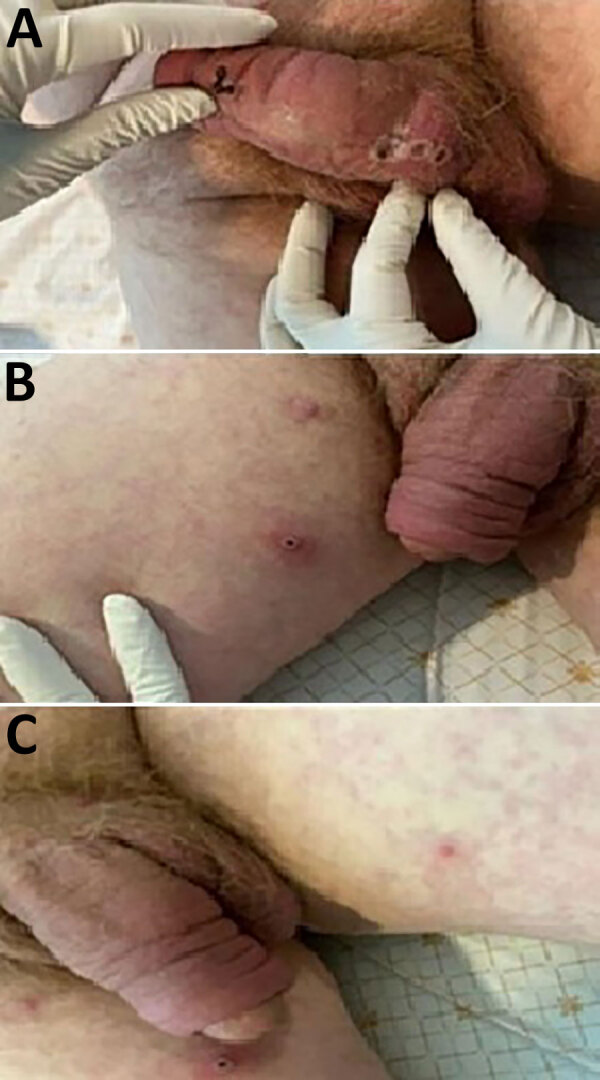
Lesions on patient who had clade Ib monkeypox virus infection linked to travel to the Democratic Republic of the Congo, Thailand, 2024. A, B) Necrotic papules with overlying scabs on the patient’s penis (A) and right thigh (B). C) Maculopapular rash and a pustule on left thigh.

We collected clinical samples on August 16, 21, and 29 and September 2. A private laboratory conducted real-time PCR and detected MPXV in the samples by amplifying the *F3L* gene; however, the result for clade I MPXV *D14L* gene amplification was inconclusive. The Thai Red Cross Emerging Infectious Disease Clinical Center also detected MPXV but did not specify the clade. We sent a swab sample from August 21 to the Bamrasnaradura Infectious Disease Institute, where clade II MPXV was identified by using the QIAstat-Dx Viral Vesicular Panel (QIAGEN, https://www.qiagen.com). Because of conflicting results, we sent a swab sample from August 16 to Thailand’s national reference laboratory at the National Institute of Health (NIH) for confirmation by whole-genome sequencing. The Thailand NIH confirmed the sample was clade Ib MPXV and deposited the sequence in the GISAID database (https://www.gisaid.org; accession no. EPI_ISL_19350788). 

The lowest cycle threshold (Ct) value of 13.73 was obtained from a sample of combined vesicles and pustules collected from the patient’s genitalia 6 days after symptom onset. The patient began treatment with tecovirimat on August 20. A subsequent swab sample from a scab lesion on the left leg showed the highest PCR Ct value 23 days after symptom onset (Ct 38.48). Cultures from all swab samples tested at NIH showed no virus growth (Table 1). The patient was discharged on September 5 without complications.

**Table 1:** Diagnostic laboratory results for swab samples from index patient who had clade Ib monkeypox virus infection linked to travel to the Democratic Republic of the Congo, Thailand, 2024*

Sample no.	Laboratory	Collection point, lesion type	No. days†	Laboratory results according to method		
				Real-time PCR (Ct)	WGS	Culture
1	Private laboratory	Genitalia, combined vesicle and pustule	6	MPXV *F3L* gene detected (13.73); *D14L* gene, inconclusive signal for clade I, not detected for clade II	NA	NA
	TRC-EIDCC			MPXV detected (15.03)	NA	NA
	Thailand NIH			NA	MPXV clade Ib‡	NA
2	BIDI	Genitalia and extremities, combined vesicle, pustule, and ulcerated lesion	11	MPXV clade II detected (21.4)	NA	NA
3	Thailand NIH	Left arm, fallen-off scab	19	ND	NA	NA
		Trunk, fallen-off scab		ND		
		Right leg, beneath scab		MPXV detected (25.17)		
		Left toes, beneath scab		MPXV detected (26.07)		
		Genitalia, ulcerated lesion		MPXV detected (34.16)		
4	Thailand NIH	Left leg, fallen-off scab	23	MPXV detected (38.48)	NA	ND
		Right leg, fallen-off scab		MPXV detected (30.88)		
		Genitalia, fallen-off scab		MPXV detected (31.12)		
		Left toes, fallen-off scab, fully formed new skin layer		MPXV detected (33.37)		

*BIDI, Bamrasnaradura Infectious Disease Institute; Ct, cycle threshold; MPXV, monkeypox virus; NA, not applicable; ND, not detected; NIH, National Institute of Health; TRC-EIDCC, Thai Red Cross Emerging Infectious Disease Center. †Number of days after onset of symptoms. ‡GISAID accession no. EPI_ISL_19350788 (https://www.gisaid.org).

Contact tracing identified 89 persons who had direct contact with the patient’s skin, bodily fluids, or contaminated objects (fomites) or who were within 1 meter of the patient during potential aerosol-generating activities. Among those 89 contacts, 33 were classified as high-risk because of exposure without proper personal protective equipment: the patient’s wife, 13 flight passengers, 12 healthcare personnel, 6 hotel staff, and 2 restaurant staff (Table 2). Symptoms did not develop in any high-risk contacts during the monitoring period; no secondary cases were observed. Because of the close contact, a regional public health officer collected nasopharyngeal swab samples from the patient’s wife on days 7, 14, and 23 after her last exposure to the patient. All samples were negative for MPXV by PCR.

**Table 2:** Characteristics of contacts of index patient who had clade Ib monkeypox virus infection linked to travel to the Democratic Republic of the Congo, Thailand, 2024*

Contacts, n = 89	No. contacts	Symptoms†	Contact level‡	Remark
Wife	1	No	High	Close contact with no PPE
Aircraft and airport				
Passenger of 2nd airline	13	No	High	Could not obtain information for first airline
Aircrew of 2nd airline	25	No	Low	
Airport officer	2	No	Low	
Private hospital				
Physician	3	No	High, n = 2; low, n = 1	No proper PPE
Nurse and nurse aide at ward A	16	No	Low	No proper PPE
Nurse and nurse aide at ward B	9	No	High	
Assistant officer	3	No	Low	
Hotel R				
Hotel officers, including housekeeper, bellboy, receptionist	6	No	High	No PPE
Elevator passenger	3	No	Low	Cannot contact
Other hotel guests in same room	6	Unknown	Unknown	Cannot contact
Restaurant A	2	No	High	No PPE

*PPE, personal protective equipment. †Symptoms as of September 5, 2024. ‡Contact risk levels: low, proper PPE; high, without proper PPE.

Before the WHO public health emergency declaration, Thailand did not have specific mpox screening measures at points of entry. Mpox cases could potentially be identified through existing yellow fever screening by the Port and Quarantine Office, which focuses on travelers from 42 yellow fever–risk countries (*5*). Screening procedures conducted by public health officers include taking travel history, checking body temperature, and verifying the International Certificate of Vaccination for yellow fever and Thailand Health Pass registration. At the time of the WHO declaration, 5 ongoing mpox outbreaks in DRC, Burundi, Kenya, Cote d’Ivoire, and Uganda overlapped with the yellow fever list. However, Rwanda was not among those. Nonetheless, on the day of arrival in Thailand, the patient voluntarily walked to the screening area wearing a long-sleeved shirt and hat and did not report any illness. Thus, the officer did not observe any visible rashes on his face.

At hotel R, public health officers from the Health Department of the Bangkok Metropolitan Administration collected environmental samples from suspected contact surfaces in the hotel room 9 days after the patient had left. All samples tested positive for MPXV except those taken from the light switch and the doorknobs of the bedroom and living room. The positive samples had Ct values ranging from 28 to 38; the lowest Ct value was detected on the curtain knob (Table 3). The hotel staff used 3 types of cleaning products containing active ingredients, such as citric acid, alkyl alcohol ethoxylate, didecyldimethylammonium chloride, alkyldimethylbenzylammonium chloride, and ethanol. At restaurant A, ethyl alcohol spray was used for table cleaning. All of those chemicals are considered effective against MPXV (*6*).

**Table 3:** Real-time PCR results of environmental samples from hotel room of index patient who had clade Ib monkeypox virus infection linked to travel to the Democratic Republic of the Congo, Thailand, 2024*

Sample no.	Collection site	MPXV PCR result	Cycle threshold value
1	Refrigerator handle	Detected	35.48
2	Curtain knob	Detected	28.64
3	Sofa	Detected	38.84
4	Bed edge	Detected	37.64
5	Air conditioner and television remote, pooled sample	Detected	35.23
6	Bathroom faucet	Detected	38.70
7	Handrail in bathroom	Detected	36.69
8	Bathtub drain	Detected	36.95
9	Light switch in bedroom	Not detected	NA
10	Doorknob in bedroom	Not detected	NA
11	Doorknob in living room	Not detected	NA

*Samples were taken 9 days after patient checked out of hotel room. MPXV, monkeypox virus; NA, not applicable.

## Conclusions

We report a travel-associated case of imported clade Ib mpox infection in Thailand with a mild clinical course, consistent with other clade Ib mpox infections reported outside of Africa (*7*). The likely source of infection was human-to-human transmission during community activities in DRC, differing from reports of predominantly sexual transmission among adults in the country (*8*,*9*). Inconsistent PCR results were attributed to the use of different detection methods across laboratory centers. Clade Ib MPXV contains a large deletion of ≈1,000 nt in the *D14L* gene region (*8**,**9*), which was the target sequence used in the private laboratory's real-time PCR and resulted in an inconclusive signal for clade I MPXV. Furthermore, the multiplex PCR kit used by the Bamrasnaradura Infectious Disease Institute exhibited cross-reactivity between MPXV clades I and II, leading to sample misidentification as clade II MPXV. The assay provider (QIAGEN) has since corrected this issue. For more accurate and timely diagnosis of clade Ib mpox, newly developed PCR methods are recommended (*10*). Swab samples taken from scab lesions still had detectable MPXV by real-time PCR; Ct values ranged from 30.88 to 38.48. Patient isolation duration and the contact monitoring period for clade Ib mpox should align with the US Centers for Disease Control and Prevention’s recommendations (*11*). 

Strengthening Thailand’s public health response is crucial to prevent future travel-associated and imported clade I mpox cases. Point-of-entry screening should include visual inspection of travelers arriving from countries facing ongoing mpox outbreaks to detect rashes on the face and extremities. Healthcare providers should consistently use appropriate personal protective equipment and obtain detailed travel history from patients manifesting clinical symptoms compatible with mpox. In 2024, mpox vaccines were not publicly available in Thailand; thus, preexposure vaccination should be considered, especially for high-risk persons.
